# Prenatal Secondhand Smoke Exposure and Infant Birth Weight in China 

**DOI:** 10.3390/ijerph9103398

**Published:** 2012-09-26

**Authors:** Nora L. Lee, Jonathan M. Samet, Gonghuan Yang, Maigeng Zhou, Jie Yang, Adolfo Correa, Peter S. J. Lees

**Affiliations:** 1 Department of Epidemiology, Bloomberg School of Public Health, Johns Hopkins University, Baltimore, MD 21205, USA; Email: jsamet@usc.edu; 2 Department of Epidemiology and Biostatistics, School of Public Health, Drexel University, Philadelphia, PA 19102, USA; 3 Department of Preventive Medicine, Keck School of Medicine and USC Institute for Global Health, University of Southern California, Los Angeles, CA 90089, USA; 4 Chinese Center for Disease Control and Prevention, Beijing 100050, China; Email: Yangghuan@vip.sina.com; 5 Center for Public Health Surveillance and Information Service, Chinese Center for Disease Control and Prevention, Beijing 100050, China; Email: maigengzhou@hotmail.com; 6 National Tobacco Control Office, Chinese Center for Disease Control and Prevention, Beijing 100050, China; Email: jlccyangjie@yahoo.com.cn; 7 Department of Medicine, University of Mississippi Medical Center, Jackson, MS 39213, USA; Email: acorrea@umc.edu; 8 Department of Environmental Health Sciences, Bloomberg School of Public Health, Johns Hopkins University, Baltimore, MD 21205, USA; Email: plees@jhsph.edu

**Keywords:** secondhand smoke, China, birth weight, pregnancy, perinatal, epidemiology

## Abstract

Epidemiologic evidence provides some support for a causal association between maternal secondhand smoke (SHS) exposure during pregnancy and reduction in infant birth weight. The purpose of this cross-sectional study is to examine the magnitude of this association in China, where both prevalence and dose of SHS exposure are thought to be higher than in U.S. populations. Women who gave birth in Beijing and Changchun September 2000–November 2001 were interviewed to quantify self-reported prenatal SHS exposure. Their medical records were reviewed for data on pregnancy complications and birth outcomes. Non-smoking women who delivered term babies (≥37 weeks gestation) were included in the study (N = 2,770). Nearly a quarter of the women (24%) reported daily SHS exposure, 47% reported no prenatal exposure, and 75% denied any SHS exposure from the husband smoking at home. Overall, no deficit in mean birth weight was observed with exposure from all sources of SHS combined (+11 grams, 95% CI: +2, +21). Infants had higher mean birth weights among the exposed than the unexposed for all measures of SHS exposure. Future studies on SHS exposure and infant birth weight in China should emphasize more objective measures of exposure to quantify and account for any exposure misclassification.

## 1. Introduction

The association between maternal active smoking during pregnancy and the risk of having a smaller baby has been well established since the 1960s based on epidemiologic studies [1,2,3]. The magnitude of this effect is, on average, a reduction of 250 grams in birth weight of babies born to smokers compared to babies born to non-smokers [4]. One large cohort study of more than 250,000 births found a 320 gram reduction in mean birth weight of infants whose mothers smoked compared to infants whose mothers did not smoke [5]. The relative risks of low birth weight with maternal prenatal smoking have ranged from 1.5 to 3.5, and an exposure-response relationship between increasing amount smoked and higher relative risk is seen in numerous studies [4]. The association between exposure to secondhand smoke (SHS) or environmental tobacco smoke (ETS) and birth weight has also been examined in epidemiologic studies, with most of these studies published within the past three decades. The body of literature includes both statistically significant and non-significant findings, but does provide some support for a causal association [6,7,8]. Relative risks and odds ratios for delivering a smaller baby for those with prenatal SHS exposure compared to no or low exposure range from 0.5 (95% CI: 0.13, 1.69) [9] to 2.31 (95% CI: 1.06, 4.99) [10], and estimated absolute reductions in mean birth weight between SHS-exposed and unexposed groups range from 3 grams [11] to 228 grams [12]. These ranges overlap somewhat with the range of measures seen with active maternal smoking, although this is expected given that it is difficult to distinguish between non-smokers highly exposed to SHS and light active smokers, with cotinine levels in these two groups being similar. It is clear, however, that the findings for prenatal SHS exposure are smaller in magnitude than findings for active maternal smoking. 

Four meta-analyses have estimated the magnitude of the effect of prenatal SHS exposure to be a mean deficit of 31–60 grams in infant birth weight [13,14,15,16]. The magnitude of this effect is weaker by a factor of 4 to 8 compared to the average 250 gram reduction in infant birth weight with active maternal smoking. Pooled odds ratios for low birth weight with SHS exposure were also reported in four meta-analyses [14,15,16,17]. These pooled overall risk estimates ranged from 1.16 to 1.60. Table 1 summarizes the five meta-analyses published over the past decade. The highest reported pooled odds ratio, 1.60, was published in Chinese [17] and the meta-analysis included both Chinese and English language studies. Liu and Chen [17] also reported pooled odds ratios for low and high levels of SHS exposure compared to no exposure: 1.53 (95% CI: 1.14, 2.04) and 2.53 (95% CI: 1.46, 4.36), respectively. These estimates overlap with the range of odds ratios reported for active maternal smoking (1.5 to 3.5 [4]), although the magnitude of effect for SHS exposure is likely smaller than for active maternal smoking during pregnancy.

**Table 1:** Meta-analyses of the impact of prenatal SHS exposure on infant birth weight.

Citation	No. studies included	Deficit in birth weight	Odds ratiofor low birth weight
Peacock *et al.* 1998 [13]	11	31 grams (95% CI: 19, 44)	-----
Windham *et al.* 1999 [14]	19	31 grams (95% CI: 20.4, 41.6)	-----
	3	-----	1.38 (95% CI: 1.01, 1.87)
Leonardi-Bee *et al.* 2008 [15]	17 prospective	33 grams (95% CI: 15.7, 51.3)	-----
	27 retrospective	40 grams (95% CI: 25.8, 54.4)	-----
	9 prospective	-----	1.32 (95% CI: 1.07, 1.63)
	17 retrospective	-----	1.22 (95% CI: 1.08, 1.37)
Liu and Chen 2009 [17] *	26	-----	1.60 (95% CI: 1.25, 2.05)
Salmasi *et al.* 2010 [16]	76	60 grams (95% CI: 39, 80)	1.16 (95% CI: 0.99, 1.36)

* Findings as reported in English language abstract.

The ability to detect a smaller magnitude of effect of prenatal SHS exposure on infant birth weight can be improved when exposure prevalence is higher, as it is in China. The prevalence of smoking among men, and consequently the prevalence of SHS exposure among women, is significantly higher in China than in the U.S. For this reason, and the fact that birth outcomes are of particular interest in a country where the one-child-per-couple family planning policy focuses attention on prenatal and newborn health, we examined the impact of prenatal SHS exposure on infant birth weight in northeast China. The primary aim of our study was to determine the difference in mean birth weight between infants whose mothers were exposed to SHS and infants whose mothers were not exposed in the Chinese cities of Beijing and Changchun (the capital of Jilin Province). A secondary aim of our study was to better characterize the SHS exposure profile in China, an important task in itself given the evidence for multiple adverse health effects associated with SHS [6,7] and the gender difference in smoking prevalence in China—63% among men and 4% among women [18]. This is in contrast to the adult smoking prevalences in the United States—24% among men and 18% among women [19]. These differences in smoking prevalence rates, and inter-country variations in public policies on cigarette smoking [20], suggest that SHS exposure patterns in China may differ from the U.S. and other developed countries. 

## 2. Methods

### 2.1. Study Population

Study participants were recruited from four major hospitals in northeast China: two in Beijing and two in Changchun. Postpartum women who had given birth to full-term (≥37 weeks gestation), live-born singletons, resided in either Beijing or Changchun, and reported never having smoked were eligible to participate in the study. Study-trained hospital health care staff (*i.e.*, physicians, nurses, and health service workers) invited new mothers into the study during their standard one-week postpartum stay in the hospital. Interviews were completed for 3,007 new mothers, but 11 were excluded due to missing data on delivery status and/or smoking status and six were excluded for multibirth deliveries. Of the remaining 2,990 women, 101 were excluded for preterm delivery, another 101 who reported smoking previously were excluded, as were 18 who reported that they were current smokers. The final study population consisted of 2,770 never-smoking women (1,363 in Beijing and 1,407 in Changchun) who had given birth in one of the four study hospitals between September 2000 and November 2001.

### 2.2. Data Collection

Following completion of informed consent and enrollment, the health care staff administered an interview to the study participant in her hospital room 1–3 days postpartum. Women were asked about their prenatal SHS exposure sources and duration, sociodemographic factors, and their knowledge and attitudes regarding SHS exposure. Specifically, for each trimester, study participants were asked to estimate their duration of exposure to SHS (as average hours per day) in the home from smoking by the husband, other family members and visitors, in the workplace from smoking by co-workers, and in public places. Additional questions were asked about the number of days per week the mother was exposed to SHS from any source, and the number of cigarettes per day the husband smoked at home. The participant’s hospital medical records were abstracted by the health care staff for data on maternal characteristics, pregnancy complications, and neonatal outcomes. The data collection forms are provided in supplementary materials.

The study was approved by the Institutional Review Board of the Johns Hopkins Bloomberg School of Public Health, the Ethical Committee of the Chinese Academy of Preventive Medicine in Beijing, and the Ethical Committee of the School of Preventive Medicine, Norman Bethune University of Medical Sciences, in Changchun.

### 2.3. Statistical Analyses

Initial analyses to examine changes in exposure prevalence and duration across trimesters consisted of testing for homogeneity (chi-square) and repeated ANOVA (F value). Since neither group level nor individual level changes in SHS exposure across trimester were significant, analyses were restricted to third trimester exposure data. Fetal growth in weight occurs mainly during the third trimester [21], and previous studies have indicated that third trimester exposure to *active* maternal smoking is associated with lower birth weight [22,23,24]. In addition, some studies have found that smoking cessation by the third trimester eliminates the risk of having a smaller baby compared to non-smokers [24,25,26]. 

The categories of sources of SHS exposure were: (1) husband smoking in the home; (2) smoking by other family members living in the home; (3) smoking by visitors to the home, or participant visiting other people’s homes where there was smoking; (4) co-workers smoking in the workplace; and (5) people smoking in public areas. The duration of exposure from each source was quantified as average hours per day, and husband’s smoking was additionally quantified as average number of cigarettes smoked per day in the home. The average daily exposure durations from all sources were summed to derive the total daily exposure to SHS, quantified as average hours per day. Women with missing data from any single source were excluded from this calculation and assigned a missing value for the total daily SHS exposure measure. Similarly, daily home exposure to SHS was calculated by summing the average daily exposure duration from all home sources (*i.e.*, husband, family members, visitors). 

Simple cross-sectional analyses of SHS exposure and infant birth weight were carried out with scatter plots for continuous data and with contingency tables for categorical data for each source of SHS exposure during the third trimester. We used unadjusted logistic regression to calculate risk estimates for demographic factors that may be associated with lower birth weight, defined in this study as <3,000 grams in birth weight [27] to increase the number of cases from 34 with low birth weight (<2,500 grams) to 328 with lower birth weight. We used cross-tabulations of demographic factors with exposure to identify potential confounders. Variables that were associated with both exposure and outcome were considered potential confounders and included in the multivariable regression models. Some variables that did not meet this criterion were nonetheless included in the models due to their strong established associations with birth weight. Multivariable adjusted linear regression models were fit to the continuous birth weight outcome and residuals from these fits were plotted to examine the validity of the assumptions of normality, linearity, and homoscedasticity. The statistical software package SAS was used for all analyses. 

## 3. Results

### 3.1. Population Characteristics

The study population consisted of 2,770 postpartum women who never smoked and gave birth to full-term (≥37 weeks gestation) live-born singleton infants. The mothers had a mean age of 27.8 years (SD = 3.5 years) and half had a university education or higher. Similarly, more than half of the fathers (54.4%) had a university education or higher. Approximately half of the mothers worked during their pregnancy, with slightly fewer working during the third trimester than the first trimester (52.4% *vs.* 55.2%, respectively). Few demographic differences were found between the two study populations in Beijing and Changchun (Table 2). Family income level did differ, despite the similarity of education levels between cities for both mothers and fathers. A larger proportion of the Beijing participants (20.7% *vs.* 6.5%) had high income levels (4,000–5,499 yuan/month) while a larger proportion of Changchun participants (55.1% *vs.* 25.0%) had lower income levels (1,000–2,499 yuan/month), closer to the national mean. Household size and house size were similar in the two cities, although one difference was significant in the home environment: 77.7% of Beijing participants had air conditioning compared to 29.7% of Changchun participants.

**Table 2:** Demographic characteristics of study population.

Characteristic	Total			Beijing		Changchun		*p*-value
	(n = 2,770)			(n = 1,363)		(n = 1,407)		
	No.	%	No.	%	No.	%		
**Maternal age at delivery**								
Mean (SD)	27.8 years (3.5)			28.9 years (3.5)		26.8 years (3.1)		<0.0001
**Maternal education level**								
Elementary school		27	1.0	15	1.1	12	0.9	0.0355 (df = 3)
Junior middle school		469	16.9	204	15.0	265	18.8	
Senior middle school		894	32.3	461	33.9	433	30.8	
University or higher		1,379	49.8	682	50.1	697	49.5	
**Paternal education level**								
Elementary school		15	0.5	6	0.4	9	0.6	0.3453 (df = 3)
Junior middle school		378	13.7	189	13.9	189	13.4	
Senior middle school		870	31.4	408	30.0	462	32.8	
University or higher		1,506	54.4	759	55.7	747	53.1	
**Family monthly income (yuan)**								
<500		36	1.4	20	1.6	16	1.1	<0.0001 (df = 5)
500–999		160	6.0	50	3.9	110	7.9	
1,000–2,499		1,089	40.8	319	25.0	770	55.1	
2,500–3,999		761	28.5	375	29.4	386	27.6	
4,000–5,499		355	13.3	264	20.7	91	6.5	
≥5,500		271	10.1	246	19.3	25	1.8	
**Household size**								
1 person (live alone)		8	0.3	7	0.5	1	0.1	0.0008 (df = 3)
2 people		1,614	58.5	820	60.4	794	56.7	
3–4 people		915	33.2	409	30.1	506	36.1	
5 or more people		222	8.1	122	9.0	100	7.2	
**Home environment**								
Average number of rooms in house (SD)		3.3 rooms (1.4)		3.1 rooms (1.3)		3.5 rooms (1.4)		<0.0001
Air conditioning		1,470	1,470	1,053	77.7	417	29.7	<0.0001 (df = 1)
Gas heating		2,562	2,562	1,229	90.2	1,333	94.8	<0.0001 (df = 1)
Gas for cooking		2,632	2,632	1,306	96.0	1,326	94.4	0.0424 (df = 1)
**Worked while pregnant**								
1st trimester		1,599	57.9	790	58.1	809	57.6	0.7693 (df = 1)
2nd trimester		1,576	57.0	770	56.7	806	57.4	0.7073 (df = 1)
3rd trimester		1,527	55.3	737	54.3	790	56.2	0.3011 (df = 1)

### 3.2. Perinatal Characteristics and Birth Outcomes

Beijing and Changchun study participants were similar in their reproductive and perinatal experiences (Table 3), with the exception that more women in Changchun were primigravida (64.3%) compared to women in Beijing (43.6%). The most common complication during pregnancy in this study population was vaginal bleeding (18.8%), followed by gestational hypertension (11.2%). Gestational diabetes and preeclampsia were not common (5.3% and 1.3%, respectively). Birth outcomes were very similar in the two cities, with mean birth weight being 57 grams higher in Beijing (3,480.0 grams, SD = 468.2) than in Changchun (3,422.9 grams, SD = 427.7). Mean birth weight was 3,451.0 grams (SD = 448.9) in this study population, and low birth weight (<2,500 grams) at term was approximately 1% in both cities, similar to the occurrence rate of 1.5% found in Qingdao, China [28]. The male/female ratio at birth, however, was higher in Changchun (1.10) than Beijing (1.01).

**Table 3:** Reproductive and perinatal characteristics of deliveries.

Characteristic	Total		Beijing		Changchun		*p*-value
	(n = 2,770)		(n = 1,363)		(n = 1,407)		
	No.	%	No.	%	No.	%	
**Prepregnancy BMI**							
Mean (SD)	20.9 (2.6)		21.1 (2.8)		20.6 (2.4)		<0.0001
**Pregnancy weight gain**							
Mean (SD)	18.4 kg (5.5 kg)		17.7 kg (5.5 kg)		19.2 kg (5.4 kg)		<0.0001
**Previous pregnancies**							
Yes	1,266	45.9	765	56.4	501	35.7	<0.0001 (df = 1)
No	1,495	54.2	592	43.6	903	64.3	
**Previous births**							
Yes	192	7.0	114	8.4	78	5.6	0.0033 (df = 1)
No	2,569	93.1	1,243	91.6	1,326	94.4	
**Pregnancy complications**							
Hypertension	308	11.2	121	9.0	187	13.3	0.0003 (df = 1)
Preeclampsia	33	1.2	25	1.8	8	0.6	0.0022 (df = 1)
Vaginal bleeding	519	18.8	265	19.5	254	18.1	0.3524 (df = 1)
Gestational diabetes	145	5.3	73	5.4	72	5.2	0.7943 (df = 1)
**Spontaneous labor and vaginal delivery**							
Yes	1,248	46.0	576	43.5	672	48.4	0.0114 (df = 1)
No	1,464	54.0	747	56.5	717	51.6	
**Infant gender**							
Male	1,419	51.4	683	50.3	736	52.4	0.2635 (df = 1)
Female	1,343	48.6	675	49.7	668	47.6	
**Infant low birth weight (<2,500 grams)**							
Yes	34	1.2	19	1.4	15	1.1	0.4333 (df = 1)
No	2,736	98.8	1,344	98.6	1,392	98.9	
**Infant birth weight**							
Mean (SD)	3,480.0 g		3,480.0 g		3,422.9 g		0.0008 (df = 1)
	(468.2 g)		(468.2 g)		(427.7 g)		

### 3.3. Risk Factors for Lower Infant Birth Weight

Although low birth weight was rare in this study population, the risk of having a lower weight baby (<3,000 grams) was increased by several maternal and perinatal factors (Table 4). Younger (≤24 years) maternal age increased the risk for a smaller baby when compared to mothers 25–29 years of age (OR for ≤24 years = 1.37, 95% CI: 1.03, 1.81). Two indicators of socioeconomic status were associated with infant birth weight: parental education and family income level. Parents with less than a university-level education were more likely to have a smaller baby, compared to parents with a university-level education (OR 1.60, 95% CI: 1.23, 2.07). The finding for family income level was consistent with the finding for education level: lower annual income level was associated with delivering a smaller baby, compared to an annual income of at least 4,000 yuan (<1,000 yuan OR 1.24, 95% CI: 0.86, 1.78; 1,000–2,499 yuan OR 1.24, 95% CI: 0.91, 1.51). 

**Table 4:** Unadjusted odds ratios for associations between parental and perinatal factors and lower infant birth weight (<3,000 grams).

Characteristic	n	Odds Ratio	95% CI
**Maternal age at delivery**			
≤24 years	568	1.37	1.03, 1.81
25–29 years	1,592	1.00	Reference
30–34 years	498	0.99	0.72, 1.37
≥35 years	110	1.07	0.59, 1.95
**Maternal height**			
1st Quartile: 142–158 cm	702	2.87	2.03, 4.06
2nd Quartile: 159–161 cm	725	1.74	1.20, 2.51
3rd Quartile: 162–164 cm	670	1.32	0.90, 1.95
4th Quartile: 165–180 cm	673	1.00	Reference
**Maternal education level**			
Junior middle school or lower	496	1.50	1.09, 2.06
Senior middle school	894	1.72	1.33, 2.23
University or higher	1,379	1.00	Reference
**Paternal education level**			
Junior middle school or lower	393	1.68	1.22, 2.31
Senior middle school	870	1.20	0.93, 1.56
University or higher	1,506	1.00	Reference
**Parental education level (combined)**			
Both less than university level	1,074	1.60	1.23, 2.07
One with university level	504	1.38	0.99, 1.91
Both with university level	1,190	1.00	Reference
**Family income**			
<1,000 yuan	196	1.20	0.74, 1.96
1,000–2,499 yuan	1,089	1.24	0.91, 1.69
2,500–3,999 yuan	761	0.99	0.71, 1.39
≥4,000 yuan	626	1.00	Reference
**Prepregnancy BMI**			
1st Quartile: 14.79–19.08	690	1.71	1.22, 2.39
2nd Quartile: 19.10–20.51	681	1.40	0.99, 1.98
3rd Quartile: 20.55–22.23	699	1.27	0.90, 1.80
4th Quartile: 22.27–34.21	691	1.00	Reference
**Pregnancy weight gain**			
1st Quartile: 2.0–14.2 kg	640	3.02	2.13, 4.27
2nd Quartile: 15.0–18.0 kg	723	1.94	1.35, 2.78
3rd Quartile: 18.5–21.6 kg	705	1.19	0.81, 1.76
4th Quartile: 22.0–48.0 kg	690	1.00	Reference
**Previous pregnancies**			
Yes	1,266	0.87	0.69, 1.10
No	1,495	1.00	Reference
**Previous births**			
Yes	192	0.62	0.36, 1.06
No	2,569	1.00	Reference
**Pregnancy complications**			
Hypertension	308	1.25	0.88, 1.76
Preeclampsia	33	2.43	1.09, 5.42
Diabetes	145	1.06	0.64, 1.76
Vaginal bleeding	519	1.04	0.78, 1.40
**Infant gender**			
Male	1,419	1.00	Reference
Female	1,343	1.54	1.22, 1.95
**Gestational age**			
37–39 weeks	1,319	2.26	1.75, 2.93
=40 weeks	1,065	1.00	Reference
>40 weeks	386	0.29	0.15, 0.56

Other reproductive factors, as consistent with the literature, are predictive of lower birth weight, including lower prepregnancy maternal BMI (lowest quartile *vs.* highest quartile OR 1.71, 95% CI: 1.22, 2.39) and lower pregnancy weight gain (lowest quartile *vs.* highest quartile OR 3.02, 95% CI: 2.13, 4.27). Shorter gestational age and female gender were also predictably associated with lower weight babies. 

### 3.4. SHS Exposure Profile

Table 5 shows that the majority of study participants (53.0%) reported some SHS exposure during their pregnancy, with exposure defined as being in the presence of someone who is actively smoking for more than 15 minutes each day (ranging from less than one day per week to almost every day). Using this definition of exposure, which is used in other international studies of SHS exposure, nearly 24% of mothers reported daily exposure and 47% reported no exposure during the entire pregnancy. The prevalence of daily exposure was nearly three times greater in Changchun (34.7%) than in Beijing (12.3%). However, when asked how many hours per day they were exposed to SHS from various sources, 58% of the women reported no exposure during the third trimester from any source (*i.e.*, home, work, and public places). 

**Table 5:** Self-reported daily average exposures to SHS during third trimester of pregnancy.

Exposure source and time	Total		Beijing		Changchun		*p*-value
	(n = 2,770)		(n = 1,363)		(n = 1,407)		
	No.	%	No.	%	No.	%	
**Any SHS exposure during entire pregnancy ^a^**							
Never	1,289	47.0	682	50.7	607	43.5	<0.0001 (df = 4)
<1 day/week	416	15.2	300	22.3	116	8.3	
1–3 days/week	283	10.3	141	10.5	142	10.2	
>3 days/week	102	3.7	56	4.2	46	3.3	
Every day	651	23.8	166	12.3	485	34.7	
**Any home exposure during third trimester**							
Yes	784	29.9	250	19.4	534	40.2	<0.0001 (df = 1)
**From husband smoking at home**							
Never	2,116	77.3	1,180	87.5	936	67.4	<0.0001 (df = 3)
<0.5 h/day	194	7.1	34	2.5	160	11.5	
0.5–0.9 h/day	142	5.2	54	4.0	88	6.3	
≥1.0 h/day	286	10.5	81	6.0	205	14.8	
None	2,054	75.1	1,125	83.5	929	67.0	<0.0001 (df = 3)
1–5 cig/day	241	8.8	123	9.1	118	8.5	
6–10 cig/day	227	8.3	58	4.3	169	12.2	
≥11 cig/day	212	7.8	41	3.0	171	12.3	
**From the workplace**							
Did not work	1,236	47.6	621	47.5	615	47.8	<0.0001 (df = 3)
Never	929	35.8	515	39.4	414	32.1	
<0.5 h/day	79	3.0	30	2.3	49	3.8	
0.5–0.9 h/day	96	3.7	52	4.0	44	3.4	
≥1.0 h/day	256	9.9	90	6.9	166	12.9	
**From public areas**							
Never	2,264	89.0	1,119	86.6	1,145	91.5	<0.0001 (df = 3)
<0.5 h/day	98	3.9	46	3.6	52	4.2	
0.5–0.9 h/day	99	3.9	80	6.2	19	1.5	
≥1.0 h/day	83	3.3	47	3.6	36	2.9	
**All home sources**							
Never	1,838	70.1	1,042	80.7	796	59.9	<0.0001 (df = 3)
≤0.5 h/day	363	13.8	114	8.8	249	18.7	
0.6–1.0 h/day	175	6.7	63	4.9	112	8.4	
>1.0 h/day	246	9.4	73	5.7	173	13.0	
**All exposure sources**							
Never	1,391	58.1	809	66.2	582	49.7	<0.0001 (df = 3)
≤0.5 h/day	369	15.4	177	14.5	192	16.4	
0.6–1.9 h/day	311	13.0	125	10.2	186	15.9	
≥2.0 h/day	322	13.5	111	9.1	211	18.0	

^a^ Exposure defined as being around someone who is actively smoking for more than 15 minutes a day.

Overall the most common source of SHS exposure during pregnancy was husband’s smoking in the home, with 22.8% of participants reporting some exposure during the third trimester. This particular exposure source was more common in Changchun (32.6%) than in Beijing (12.5%). In addition, a larger proportion of Changchun mothers (12.3%) reported that their husbands smoked more than half a pack of cigarettes per day in the home compared to Beijing mothers (3.0%). Conversely, a larger proportion of women in Beijing (83.5%) reported no exposure from husbands smoking in the home compared to women in Changchun (67.0%). Combining both cities, approximately 75% of the mothers reported never being exposed to husbands smoking at home during the third trimester.

The second most common source of SHS exposure overall was the workplace: 16.6% of the study population experienced exposure in the workplace (calculated from Table 5), although nearly half the women did not work during their third trimester. Among those who did work, 31.7% reported exposure in the workplace (calculated from Table 5). Among women who had a summed total duration of SHS exposure greater than zero hours per day, proportional exposure duration from home sources, work, and public places were calculated (Table 6). Nearly half the women (48.1%) reported that their SHS exposure was entirely from the home environment, and nearly a quarter of the women (22.4%) who worked during their third trimester reported that their SHS exposure was entirely from the workplace. 

**Table 6:** SHS exposure sources relative to total SHS exposure among women with any exposure to SHS during third trimester of pregnancy (n = 1,002).

	Percent of women with any SHS exposure		
Proportion of hours/day of total SHS exposure	Home	Public places	Workplace *
None	26.2	74.5	37.4
1%–24% of total SHS exposure	5.5	7.3	2.8
25%–49% of total SHS exposure	6.6	5.1	10.6
50%–74% of total SHS exposure	10.3	5.2	16.7
75%–99% of total SHS exposure	3.4	0.2	10.1
100% of total SHS exposure	48.1	7.8	22.4

* Among women who worked during the third trimester (n = 604).

The SHS exposure profile varied by several demographic characteristics in this population (Table 7). An increasing proportion of women reported any SHS exposure with decreasing age. Thus, a greater proportion of younger women (≤24 years of age) reported SHS exposure than women 35 years and older (48.2% *vs.* 25.8%). This age trend was consistent for all categories of SHS exposure duration (*p* = 0.0032; df = 9). Fewer women with university level educations who were married to men with university level educations reported any SHS exposure during pregnancy (37.8%) compared to couples for whom neither spouse had a university level education (46.3%). This difference in parental education level was evident across all categories of SHS exposure duration (*p* = 0.0089; df = 6). Family income level, which is correlated with parental education level, showed a similar trend. 

**Table 7:** Percentage distribution of daily average SHS exposure times during third trimester of pregnancy by parental and perinatal characteristics.

Characteristic	Total average daily SHS exposure during third trimester from all sources (hours/day)					
	n	Never	<0.5	0.6–1.9	>2.0	*p*-value
**Maternal age at delivery**		(%)	(%)	(%)	(%)	0.0032 (df = 9)
≤24 years	485	51.8	17.3	14.4	16.5	
25–29 years	1,362	57.8	16.2	12.9	13.1	
30–34 years	448	62.7	12.7	12.5	12.1	
≥35 years	97	74.2	7.2	8.3	10.3	
**Maternal height**						
1st Quartile: 142–158 cm	596	57.9	16.1	12.1	13.9	0.2299 (df = 9)
2nd Quartile: 159–161 cm	631	58.2	14.7	14.4	12.7	
3rd Quartile: 162–164 cm	582	57.9	13.4	12.4	16.3	
4th Quartile: 165–180 cm	584	58.6	17.5	13.0	11.0	
**Maternal education level**						
Junior middle school or lower	446	50.9	17.3	13.9	17.9	0.0074 (df = 6)
Senior middle school	768	58.1	15.4	12.6	13.9	
University or higher	1,179	60.9	14.8	12.9	11.5	
**Paternal education level**						
Junior middle school or lower	351	50.1	15.7	14.5	19.7	0.0002 (df = 6)
Senior middle school	760	55.3	17.4	14.0	13.4	
University or higher	1,282	62.0	14.2	12.0	11.8	
**Parental education level (combined)**						
Both less than university level	944	53.7	17.0	13.7	15.7	0.0089 (df = 6)
One with university level	437	58.4	14.2	13.3	14.2	
Both with university level	1,012	62.2	14.5	12.3	11.1	
**Family monthly income**						
<1,000 yuan	154	54.6	13.0	12.3	20.1	0.0046 (df = 9)
1,000–2,499 yuan	915	55.3	15.1	15.3	14.3	
2,500–3,999 yuan	674	57.3	16.3	12.8	13.7	
≥4,000 yuan	569	64.0	15.8	9.8	10.4	

### 3.5. SHS Exposure and Birth Weight

Multiple sources and levels of exposure to SHS were examined in relation to infant birth weight. The adjusted mean differences in birth weight by SHS source and by city are presented in Table 8. Overall, mean infant birth weight did not differ significantly between mothers exposed and not exposed to SHS during the third trimester of pregnancy. Similarly, most of the differences in mean infant birth weight associated with various measures and various sources of self-reported SHS exposure were not statistically significant. The exception was an increase of 49 grams (95% CI: +19, +78) for each hour per day of self-reported exposure to SHS in public places. The other exposure sources were associated with slight increases in adjusted mean birth weights, ranging from 2 to 16 grams for each hour per day of SHS exposure. 

When examined by city, infants exposed in Beijing were on average 32 grams heavier than unexposed infants in Beijing (95% CI: −20, +84). As with the overall finding with exposure to SHS in public places, infants in Beijing were heavier by 47 grams for each hour per day of exposure to SHS in public places, and this finding was also statistically significant (95% CI: +11, +82). A slight increase in mean infant birth weight that was statistically significant was also seen for the sum of exposures from all sources (18 grams for each hour per day of exposure, 95% CI: +3, +34). No statistically significant effect of SHS on mean birth weight was found among infants in Changchun with the various sources and measures of self-reported SHS exposure.

**Table 8:** Adjusted ^a^ mean differences in birth weight for self-reported exposures to SHS during third trimester of pregnancy, by city.

	All			Beijing			Changchun		
	Diff.			Diff.			Diff.		
	N	(grams)	(95% CI)	N	(grams)	(95% CI)	N	(grams)	(95% CI)
Any exposure during entire pregnancy ^b^ (yes/no)	2,627	+16	(−17, +48)	1,251	+32	(−20, +84)	1,376	+3	(−39, +46)
Sum of exposures from all sources (hours/day)	2,296	+11	(+2, +21)	1,142	+18	(+3, +34)	1,154	+5	(−7, +18)
Sum of exposures from home sources (hours/day)	2,517	+11	(−2, +24)	1,207	+15	(−3, +33)	1,310	+5	(−14 +23)
Workplace exposure (hours/day)	2,483	+4	(−12, +19)	1,215	0	(−26, +27)	1,268	+4	(−15, +24)
Public place exposure (hours/day)	2,443	+49	(+19, +78)	1,208	+47	(+11, +82)	1,235	+52	(−2, +106)
Exposure from husband smoking at home (hours/day)	2,624	+10	(−10, +30)	1,255	+15	(−13, +43)	1,369	+2	(−27, +31)
Exposure from husband smoking at home (cigarettes/day)	2,619	+2	(−1, +4)	1,252	+6	(0, +12)	1,367	+1	(−2, +3)

^a^ Adjusted for maternal age, maternal education, family income, maternal body mass index, maternal weight gain during pregnancy, gestational age, gender. ^b^ Exposure defined as being around someone who is actively smoking for more than 15 minutes a day and for one or more days per week during the pregnancy.

### 3.6. Sensitivity Analysis

A sensitivity analysis was carried out to assess the potential impact of exposure misclassification, specifically among those reporting no SHS exposure. Up to 45% of the self-reported unexposed group were randomly selected and reclassified equally as being exposed at the low (≤0.5 hours/day), middle (0.6–1.9 hours/day), and high (≥2.0 hours/day) exposure groups and the full linear regression model was run again. The random selection, reclassification, and regression were repeated 1,000 times in a simulation to obtain means of the difference in birth weight, standard error, and p-value. Compared to the findings from the empirical data (0% reclassified), the findings from the simulation when 15%, 30%, and 45% were reclassified as exposed were not notably altered (Table 9). However, if social desirability response bias is present, then exposure misclassification would be greater among women who reported no home exposures. When these women were excluded from the linear regression analysis, those with daily prenatal SHS exposure (more than 15 minutes each day) had babies that were on average 125 grams (95% CI: −250.8, +0.5) lighter than babies with less than one day per week of prenatal exposure to SHS. 

**Table 9:** Repeated random reclassification (1,000) of self-reported unexposed group to various exposure levels: Means of adjusted differences in mean birth weights with SHS exposure after repeated reclassification.

Proportion of apparently unexposed randomly reclassified to exposed	Exposure group *	Summary of parameter estimates		
		Mean of difference in mean birth weights relative to unexposed (g)	Mean	Mean
			standard error of mean difference (g)	*p*-value
0% (original data without reclassification)	Low	−0.7	21	0.9
	Medium	+30	23	0.2
	High	+37	23	0.1
15% (5% to low, 5% to med, 5% to high)	Low	−0.2	20	0.7
	Medium	+24	22	0.3
	High	+31	21	0.2
30% (10% to low, 10% to med, 10% to high)	Low	−0.1	20	0.7
	Medium	+22	21	0.4
	High	+26	21	0.3
45% (15% to low, 15% to med, 15% to high)	Low	+0.4	20	0.6
	Medium	+19	21	0.4
	High	+23	21	0.3

* Low ≤ 0.5 h/day; Medium 0.6−1.9 h/day; High ≥ 2.0 h/day.

## 4. Discussion

Overall, we found no association between prenatal SHS exposure and birth weight among babies after taking into account the effects of known predictors of birth weight. Few studies in the published literature examine prenatal SHS exposure and infant birth weight in China. Five such studies conducted in China can be found through PubMed—three in English [29,30,31] and two in Chinese with English abstracts [32,33]. In a cross-sectional study of 1,058 infants, Chen *et al.* [29] reported a decrease of 11 grams in the average birth weight of exposed infants (3,242 grams, SD = 557) compared to unexposed infants (3,253 grams, SD = 430). Pan [32] found a 1.7 odds ratio (calculated 95% CI: 0.69, 4.10) for small-for-gestational-age (SGA) with husbands smoking at home compared to unexposed (n = 253). In a cross-sectional study of 1,785 infants, however, Zhang and Ratcliffe [30] found a slight increase of 32 grams in average birth weight among infants whose fathers smoked a pack a day or more (3,213 grams, 95% CI: 3,025, 3,401) compared to fathers who did not smoke (3,191 grams, 95% CI: 2,995, 3,367). In a prospective study of 1,388 mothers who self-reported SHS exposure during pregnancy, mean birth weight of infants among exposed mothers was 30.5 grams less (*p* = 0.2327) than the mean birth weight of infants whose mothers were not exposed [31]. These four studies were conducted in or near Shanghai, while the matched case-control study of 310 infants by Han *et al.* [33] was conducted in Beijing. The Beijing study reported the only statistically significant finding—an odds ratio of 3.42 (95% CI: 1.44, 8.14; *p* < 0.01) for SGA with SHS exposure during pregnancy. In our study, average infant birth weight was actually lowest among the unexposed group compared to every other category of exposure level as measured by cigarettes per day smoked by the husband. The average birth weight does decrease, however, with increasing level of SHS exposure among the exposed groups, suggesting possible misclassification of the self-reported unexposed group.

Stratification by city revealed a slight positive and statistically significant association between self-reported SHS exposure and birth weight in Beijing, with exposed women having slightly heavier newborns than unexposed women, while no statistically significant associations were found among the Changchun study participants. The main contribution to SHS exposure in this population was from the home environment, and specifically the husband smoking in the home, although self-reported SHS exposure among women in this study was not consistent with male smoking rates and did not follow the same age-prevalence pattern for male smokers, as described in a report of a 1996 national survey conducted in China [18]. In our study population, 75% of the women denied any SHS exposure from the husband smoking at home and 58% reported no exposure from any sources (*i.e.*, home, work, public places), yet the 1996 national survey finds that 64% of urban men in China are active smokers. Another national study conducted in China in 2000–2001 reported an active smoking prevalence of 54.5% among urban men [34]. This inconsistency between self-reported SHS exposure prevalence in our study and reported active smoking rate among urban men in China may reflect underreporting of exposure in our study.

Underreporting of SHS exposure during pregnancy may have occurred due to social desirability response bias, a phenomenon that has been examined and reported in other areas of research [35,36,37]. Such bias has been defined as a tendency to provide responses that deny socially undesirable traits [37], or that are consistent with societal beliefs [36]. Given the family planning policy in China, there is high social and psychological value ascribed to having a healthy (and large) baby. At the time of this study, Beijing had an anti-smoking public health campaign, as well as provided prenatal care that incorporated education about SHS exposure. The public health campaign, which began in 1987, included television and newspaper coverage of anti-smoking messages, banning smoking in public places (such as the Beijing Railway Station), and banning cigarette advertising within the city [38]. The national Chinese Association on Smoking and Health (CASH) was established in 1990 (now the Chinese Association on Tobacco Control), with a branch office in Beijing. CASH was charged with three tasks: (1) Prevent and stop smoking among young people; (2) Develop policies, laws and regulations for tobacco control; and (3) Educate and broadcast messages about the harmful effects of tobacco [38]. Changchun did not have a CASH branch office, and did not have similar programs in place. Thus, mothers in Beijing with smaller babies may have underreported their SHS exposure during pregnancy, resulting in the finding of heavier babies among those exposed in Beijing (but not in Changchun) compared to those not exposed. 

Another indication of possible exposure misclassification is that exposure prevalence and exposure duration in our study population decreased with increasing maternal age, while in the 1996 national survey population smoking prevalence among men in China increased with increasing age. Assuming that the ages of husband and wife are correlated, the reversal of age-prevalence patterns of male smoking in the survey population and SHS exposure in the study population is a notable difference. This may be driven in part by paternal education level: 53% of women 35 years and older had husbands with a university education or higher, whereas only 35% of women under 25 years of age had husbands with a university education (*p* < 0.0001). Similarly, 26% of younger women and 49% of older women had a university education. Education level, however, did not significantly modify the effect of maternal age on infant birth weight after adjusting for the known confounders, likely due to the younger women not reaching the age to have attained a university degree (48% of the youngest age category were <23 years of age).

In the 1996 national survey, 53.5% of nonsmokers reported SHS exposure for at least 15 minutes per day on more than one day per week [18]. The 2000–2001 national study reported SHS exposure among approximately 53% of nonsmoking urban women [34]. In our study, only 37.8% of new mothers reported SHS exposure for at least 15 minutes per day at least one day per week during their pregnancy. Prevalence of exposure from the various sources was also higher in the two nationally representative populations than in our study population. Among the 1996 survey sample, 71% reported exposure in the home and 25% in the workplace (all respondents, age 15–69 years). In the 2000–2001 study, 57.5% reported home exposure and 33.8% reported workplace exposure (nonsmoking women 35–44 years of age). Among our study population of new mothers, 30% reported exposure in the home and 17% in the workplace (32% among those who worked during pregnancy). The much higher overall and source-specific exposure prevalences in the national population samples suggest exposure misclassification among our study participants. 

The sensitivity analysis results showed no change in our study findings when up to 45% of the total unexposed group was reclassified as exposed, but did change our findings when women with no reported home exposures were excluded from the analysis. This sensitivity analysis showed a reduction in infant birth weight, which may reflect a greater degree of self-reported exposure misclassification from home sources than from other sources. In an effort to minimize recall bias, the new mothers were asked about their prenatal SHS exposure within a week after giving birth. Since the findings suggest, however, that bias and exposure misclassification are likely, the possibility should be considered that an awareness of the potential negative effects of SHS influenced the accuracy of self-reported exposure during pregnancy. 

The exposure indicators in this study were limited and measures of nicotine and its metabolite, cotinine, were not attainable. The mother’s self-reported exposure duration is clearly a crude estimate of actual exposures. In addition, misclassification is suggested by the discrepancies in the prevalence of SHS exposure in this study population compared to other study populations in China and the two national estimates of exposure in China. If a true association between higher prenatal SHS exposure and lower infant birth weight exists in our study population, then underreporting of SHS exposure by the mothers could have resulted in our null findings. However, the crude measures of summed daily SHS exposure duration do not account for the possibility that exposure sources may have overlapped in time, and thus may overrepresent the actual hours per day in which a woman was exposed. Such misclassification of exposure would bias the association towards the null hypothesis. Another limitation of our study is the generalizability of our findings, since the study population is exclusively urban. In addition to demographic differences between urban and rural populations, smoking and SHS exposure rates are known to be somewhat higher in rural than urban populations in China [18,39]. 

The advantage of this current study design, however, is the inclusion of SHS from all sources. Zhang and Ratcliffe [30] had data only on the number of cigarettes smoked per day by the father at the time of the study (not during the pregnancy), while Chen *et al.* [29] had data on the number of cigarettes smoked per day by the father and other family members in the home. Pan [32] also included workplace SHS exposure, which was shown in this current study to be an important source of SHS (44% of total exposure time among those who worked during pregnancy). Since more than half the women in the current study worked during their pregnancy, workplace exposures are non-trivial.

Despite the variations in SHS exposure prevalences found in this study and the other studies in China, exposure remains more prevalent in China than in the U.S. Table 10 summarizes the published studies (in English) reporting SHS exposure prevalence among women in China. The reported prevalences range from 51% [31] to 75% [40], with findings from the more recent studies in the higher end of the range. In comparison, data from the 1999–2000 National Health and Nutrition Examination Survey (NHANES) show that 52.5% of the non-smoking population in the U.S. was exposed to SHS (defined as serum cotinine level ≥0.05 ng/mL) [19]. This population exposure prevalence dropped to 40.1% in the 2007–2008 NHANES [19]. In that same time frame, SHS exposure among non-smoking women in the U.S. dropped from 47.5% to 37.4% [19]. One U.S. cohort study looked specifically at SHS exposure during pregnancy and found that 30% of mothers reported exposure in the three months before conception through pregnancy [41]. Both home and work sources of exposure were accounted for in this multi-site population of 4,667 controls enrolled in a national birth defects study from 1997 to 2003. 

The estimated incidence of low birth weight (standardly defined as <2,500 grams) in urban China is 4.2% [42] while the mean birth weight is approximately 3,300 grams [42]. The incidence of low birth weight in this urban study population was notably lower at 1.2% and the mean birth weight was slightly higher at 3,480 grams. The better birth weight outcomes in our study may be due to the study population not being representative of urban China, since Changchun is a provincial capital and Beijing is the national capital. 

**Table 10:** Published studies reporting SHS exposure prevalence among women in China.

Citation and Location	Study design	Study population	Exposure measures and indicators	Findings
Chen, *et al.* (1989) [29] Shanghai	Cross-sectional	1,058 births to non-smoking women, 1981	Self-report retrospectively	72% had a smoking family member in the home
Zhang and Ratcliffe (1993) [30] Shanghai	Control group in case-control study of birth defects	1,785 births to non-smoking women, 1986–1987	Self-report retrospectively	58% exposed to paternal smoking
Yang, *et al.* (1999) [18]	Nationally representative survey	120,298 selected from cluster random sampling, 1996	Self-report	More than 60% of non-smoking women of child-bearing age (25–50 years) exposed at least 15 minutes daily more than 1 day per week
Loke, *et al.* (2000) [43] Guangzhou	Cross-sectional	1,449 never-smoking pregnant women, 1996−1997	Self-report at time of enrollment (during pregnancy)	60.2% of women had smoking husbands and 71% of these women had SHS exposure at home; 33% of women with non-smoking husbands had SHS exposure at home
Gu, *et al.* (2004) [34]	Cross-sectional nationally representative sampling	15,540 adults, 2000−2001	Self-report	51.3% of non-smoking women had SHS exposure at home
Wu, *et al.* (2007) [31] Anqing	Cohort of textile mills workers	1,388 new mothers with births, 1996−2000	Prospective self-report	51% exposed to SHS at home during pregnancy
Fu, *et al.* (2008) [44] Shanghai	Cross-sectional	701 never-smoking new mothers with infants, 2005−2006	Retrospective self-report	41.9% exposed to SHS during pregnancy; 55.9% exposed to SHS 3 months before pregnancy
Wang, *et al.* (2009) [45] Sichuan, Jiangxi and Henan	Cross-sectional survey in 6 counties	8,142 non-smokers, 2004	Self-report	71% of non-smoking women exposed at least 15 minutes daily at least 1 day per week
Yang, *et al.* (2010) [40] Sichuan	Cross-sectional	1,181 randomly selected never-smoking pregnant women with smoking husbands, 2008	Self-report at time of enrollment (during pregnancy); subset with hair nicotine analysis	75.1% exposed at least 15 minutes daily more than 1 day per week
Xiao, *et al.* (2010) [39]	Nationally representative survey (Global Adult Tobacco Survey)	13,354 selected from cluster random sampling, Dec 2009−Mar 2010	Self-report	71.6% of women exposed to SHS
Hsia, *et al.* (2010) [46]				

## 5. Conclusions

Our findings provide no evidence that prenatal SHS exposure reduces mean infant birth weight at term, although exposure misclassification is likely to be a factor in these findings. The differences in exposure profiles between cities and the crude measures of self-reported SHS exposure in this study and the studies conducted in Shanghai warrant further investigation of the potential health impact of prenatal SHS exposure on infant birth weight at term. An exploration of the factors that contribute to the differences in findings between cities, and a more objective measure of SHS exposure, may lead to a better understanding of the risks to fetuses posed by the relatively high prevalence of smoking in China. The vast majority of SHS exposure occurs in the home and the workplace. Reducing exposure in these two environments could have an impact on birth outcomes if prenatal SHS exposure adversely affected fetal growth and development, but would also benefit the larger population in which SHS exposure is associated with multiple adverse health effects [6,7]. Future studies in China on SHS exposure and its potential health impact should emphasize more objective measures of exposure to clarify the relationship in a population where smoking is a critical public health issue.

## Supplementary Files

Supplementary File 1: PDF-Document (PDF, 59 KB)
