# Supplementary material for: Prenatal Secondhand Smoke Exposure and Infant Birth Weight in China
Source: Int J Environ Res Public Health. 2012 Sep 26;9(10):3398–420. doi: 10.3390/ijerph9103398 (PMC3509463; doi:10.3390/ijerph9103398)
Supplement: Supplementary File 1: — PDF-Document (PDF, 59 KB) [file ijerph-09-03398-s001.pdf]

OPEN ACCESS

International Journal of
Environmental Research and
Public Health
ISSN 1660-4601
www.mdpi.com/journal/ijerph

Supplementary Information

Prenatal Secondhand Smoke Exposure and Infant Birth Weight in China

Nora L. Lee [1,2,]*, Jonathan M. Samet [1,3], Gonghuan Yang [4], Maigeng Zhou [5], Jie Yang [6], Adolfo Correa [7] and Peter S. J. Lees [8]

[1] Department of Epidemiology, Bloomberg School of Public Health, Johns Hopkins University, Baltimore, MD 21205, USA; E-Mail: jsamet@usc.edu
[2] Department of Epidemiology and Biostatistics, School of Public Health, Drexel University, Philadelphia, PA 19102, USA
[3] Department of Preventive Medicine, Keck School of Medicine and USC Institute for Global Health, University of Southern California, Los Angeles, CA 90089, USA
[4] Chinese Center for Disease Control and Prevention, Beijing 100050, China; E-Mail: Yangghuan@vip.sina.com
[5] Center for Public Health Surveillance and Information Service, Chinese Center for Disease Control and Prevention, Beijing 100050, China; E-Mail: maigengzhou@hotmail.com
[6] National Tobacco Control Office, Chinese Center for Disease Control and Prevention, Beijing 100050, China; E-Mail: jlccyangjie@yahoo.com.cn
[7] Department of Medicine, University of Mississippi Medical Center, Jackson, MS 39213, USA; E-Mail: acorrea@umc.edu
[8] Department of Environmental Health Sciences, Bloomberg School of Public Health, Johns Hopkins University, Baltimore, MD 21205, USA; E-Mail: plees@jhsph.edu

* Author to whom correspondence should be addressed; E-Mail: nllee@drexel.edu; Tel.: +1-215-762-4946; Fax: +1-215-762-1174.

Appendix

DATA COLLECTION FORM

For Study on Pregnant Women and Their Newborns

Conducted By

Johns Hopkins University

Chinese Academy of Preventive Medicine

and

Norman Bethune University of Medical Sciences

DONGCHENG OBSTETRICAL HOSPITAL

Name of Interviewer: ___________________________

Subject Identification Number: __1__ __0__ ___ ___ ___
1 2 3 4 5

Patient's Medical Record Number: ___________________________

Date of Interview: ___ ___ - ___ ___ - ___ ___
24 25 26 27 28 29
year month day

BEIJING OBSTETRICAL AND GYNECOLOGICAL HOSPITAL

Name of Interviewer: ___________________________

Subject Identification Number: __2__ __0__ ___ ___ ___
1 2 3 4 5

Patient's Medical Record Number: ___________________________

Date of Interview: ___ ___ - ___ ___ - ___ ___
24 5 26 27 28 29
year month day

COOPERATIVE HOSPITAL

Name of Interviewer: ___________________________

Subject Identification Number: __3__ __0__ ___ ___ ___
1 2 3 4 5

Patient's Medical Record Number: ___________________________

Date of Interview: ___ ___ - ___ ___ - ___ ___
24 25 26 27 28 29
year month day

INTERVIEW WITH MOTHER

Section 1. Eligibility and Demographics

1. DO YOU LIVE IN BEIJING? ___ 30
   - 0. No
   - 1. Yes
   - 9. Do not know / No answer

2. DID YOU EVER SMOKE CIGARETTES? ___ 31
   - 0. No, never smoked before
   - 1. Yes, but stopped smoking before pregnancy
   - 2. Yes, but stopped smoking during pregnancy
   - 3. Yes, now smoke on average less than 1 cigarette/day
   - 4. Yes, now smoke on average 1 or more cigarettes/day
   - 9. Do not know / No answer

3. WHAT IS THE HIGHEST GRADE OR YEAR OF SCHOOL YOU HAVE COMPLETED? ___ 32
   - 0. No education / Illiterate
   - 1. Elementary school
   - 2. Junior middle school
   - 3. Senior middle school
   - 4. University and higher
   - 9. Do not know / No answer

4. WHAT IS THE HIGHEST GRADE OR YEAR OF SCHOOL YOUR HUSBAND HAS COMPLETED? ___ 33
   - 0. No education / Illiterate
   - 1. Elementary school
   - 2. Junior middle school
   - 3. Senior middle school
   - 4. University and higher
   - 9. Do not know / No answer

5. HOW MANY PEOPLE ARE IN YOUR FAMILY THAT LIVE AND EAT WITH YOU, INCLUDING YOURSELF? ___ ___ 34 35

   _____ (*Enter. one number*)

6. HOW MUCH IS YOUR FAMILY'S TOTAL MONTHLY INCOME? ___ 36
   - 0. less than 500 yuan
   - 1. 500 yuan–999 yuan
   - 2. 1,000 yuan–2,499 yuan
   - 3. 2,500 yuan–3,999 yuan
   - 4. 4,000 yuan–5,499 yuan
   - 5. 5,500 yuan or more
   - 9. Do not know / No answer

7. WHEN YOU WERE PREGNANT, HOW MANY DAYS EACH WEEK WERE YOU AROUND ANYONE FOR MORE THAN 15 MINUTES EACH DAY WHO WAS SMOKING? (*Read. choices 0–4*)

0. Never
1. Less than 1 day each week
2. 1–3 days each week
3. More than 3 days each week
4. Almost every day
9. Do not know / No answer

___ 37

Section 2. Home Exposure Levels

8. AT HOME, HOW MANY HOURS EACH DAY WERE YOU AROUND YOUR HUSBAND WHEN HE WAS SMOKING DURING YOUR . . .

. . . 1st TRIMESTER?

98. Husband did not smoke at home

Average hours per day: _____

99. Do not know / No answer

___ ___ 38 39

. . . 2nd TRIMESTER?

98. Husband did not smoke at home

Average hours per day: _____

99. Do not know / No answer

___ ___ 40 41

. . . 3rd TRIMESTER?

98. Husband did not smoke at home

Average hours per day: _____

99. Do not know / No answer

___ ___ 42 43

9. AT HOME, HOW MANY CIGARETTES DID YOUR HUSBAND SMOKE EACH DAY INSIDE YOUR HOME DURING YOUR . . .

. . . 1st TRIMESTER?

98. Husband did not smoke at home

Average number of cigarettes: _____

99. Do not know / No answer

___ ___ 44 45

. . . 2nd TRIMESTER?

98. Husband did not smoke at home

Average number of cigarettes: _____

99. Do not know / No answer

___ ___ 46 47

. . . 3rd TRIMESTER?

98. Husband did not smoke at home

Average number of cigarettes: _____

99. Do not know / No answer

___ ___ 48 49

10. AT HOME, HOW MANY HOURS EACH DAY WERE YOU AROUND YOUR OTHER FAMILY MEMBERS WHEN THEY WERE SMOKING DURING YOUR . . .

. . . 1st TRIMESTER? ___ ___ 50 51

98. Other family members did not smoke at home

Average hours per day: _____

99. Do not know / No answer

. . . 2nd TRIMESTER? ___ ___ 52 53

98. Other family members did not smoke at home

Average hours per day: _____

99. Do not know / No answer

. . . 3rd TRIMESTER? ___ ___ 54 55

98. Other family members did not smoke at home

Average hours per day: _____

99. Do not know / No answer

11. HOW MANY HOURS EACH DAY WERE YOU VISITING OTHER PEOPLE OR AROUND VISITORS OR GUESTS WHEN THEY WERE SMOKING DURING YOUR . . .

. . . 1st TRIMESTER? ___ ___ 56 57

98. They did not smoke in the homes

Average hours per day: _____

99. Do not know / No answer

. . . 2nd TRIMESTER? ___ ___ 58 59

98. They did not smoke in the homes

Average hours per day: _____

99. Do not know / No answer

. . . 3rd TRIMESTER? ___ ___ 60 61

98. They did not smoke in the homes

Average hours per day: _____

99. Do not know / No answer

12. HOW MANY ROOMS DOES YOUR HOME HAVE, INCLUDING THE KITCHEN (IF SEPARATE ROOM WITH DOOR) BUT EXCLUDING BATHROOMS AND CLOSETS? ___ ___ 62 63

_____ (*Enter. one number*)

99. Do not know / No answer

13. WHEN SOMEONE IS SMOKING IN YOUR HOME, HOW OFTEN DO YOU OPEN THE WINDOWS OR DOORS? (*Read. choices 0–5*) ___ 64

0. No smoking in the home
1. Never
2. Seldom
3. Sometimes
4. Often
5. Always
9. Do not know / No answer

14. DO YOU HAVE AIR CONDITIONING IN YOUR HOME? ___ 65

0. No
1. Yes
9. Do not know / No answer

15. WHAT TYPE OF HEATING DO YOU HAVE IN YOUR HOME? ___ 66

0. Electricity
1. Gas heating
2. Stove
3. Other (specify): ____________________ 67–84
9. Do not know / No answer

16. WHAT TYPE OF FUEL DO YOU USE MOST OFTEN FOR COOKING? ___ 85

0. Electricity
1. Natural gas
2. Wood
3. Coal
4. Other (specify): ____________________ 86–103
9. Do not know / No answer

Section 3. Work Exposure Levels

17. DID YOU WORK WHILE YOU WERE PREGNANT? ___ 104

0. No (*If "No," check "Did not work" for questions 18–21*)
1. Yes
9. Do not know / No answer

18. AT WORK, HOW OFTEN WERE YOU AROUND ANYONE FOR MORE THAN 15 MINUTES EACH DAY WHO WAS SMOKING WHEN YOU WERE PREGNANT? (*Read. choices 0–4*) ___ 105

0. Did not work
1. Less than 1 day each week
2. 1–3 days each week
3. More than 3 days each week
4. Almost every day
9. Do not know / No answer

19. ON AVERAGE, HOW MANY PEOPLE SMOKED NEAR YOU AT WORK WHEN YOU WERE PREGNANT?

98. Did not work

_____ (*Enter. one number*)

99. Do not know / No answer

___ ___ 106 107

20. WHERE DID YOU WORK MOST OF THE DAY? (*Read. choices 1–4*)

___ 108

0. Did not work
1. In an office or room with other people
   HOW MANY OTHER PEOPLE WORK IN YOUR OFFICE OR ROOM? _____ (*Enter one number*)
2. Inside a building, but did not stay in one room
3. Outside
4. Other (specify): ____________________
9. Do not know / No answer

___ ___ 109 110

111–126

21. ON AVERAGE, HOW MANY HOURS EACH WORK DAY WERE YOU AROUND SOMEONE AT WORK WHO WAS SMOKING DURING YOUR . . .

. . . 1st TRIMESTER?

98. Did not work

Average hours per day: _____

99. Do not know / No answer

___ ___ 127 128

. . . 2nd TRIMESTER?

98. Did not work

Average hours per day: _____

99. Do not know / No answer

___ ___ 129 130

. . . 3rd TRIMESTER?

98. Did not work

Average hours per day: _____

99. Do not know / No answer

___ ___ 131 132

Section 4. Public Exposure Levels

22. IN PUBLIC PLACES (NOT AT WORK) LIKE BUSES, RESTAURANTS OR PARKS, ON AVERAGE, HOW MANY HOURS EACH DAY WERE YOU AROUND SOMEONE WHO WAS SMOKING DURING YOUR . . .

. . . 1st TRIMESTER?

98. Never

Average hours per day: _____

99. Do not know / No answer

___ ___ 133 134

. . . 2nd TRIMESTER?

98. Never

Average hours per day: _____

99. Do not know / No answer

___ ___ 135 136

. . . 3rd TRIMESTER?

98. Never

Average hours per day: _____

99. Do not know / No answer

___ ___ 137 138

Section 5. Knowledge and Attitudes

23. DO YOU THINK IT WOULD BE HARMFUL TO THE BABY IF A MOTHER SMOKED WHILE SHE WAS PREGNANT? ___ 139
    - 0. No
    - 1. Yes
    - 9. Do not know / No answer

24. WHAT DO YOU THINK WOULD BE THE EFFECT ON THE BABY IF THE MOTHER SMOKED DURING PREGNANCY? ___ 140
    - 0. No effect
    - 1. Physical defect
    - 2. Mental retardation or developmental delay
    - 3. Miscarriage or low birth weight
    - 4. Cancer
    - 5. Other (specify): ____________________ 141–156
    - 9. Do not know / No answer

25. DO YOU THINK IT WOULD BE HARMFUL TO THE BABY IF OTHERS SMOKED AROUND THE MOTHER WHILE SHE WAS PREGNANT? ___ 157
    - 0. No
    - 1. Yes
    - 9. Do not know / No answer

26. WHAT DO YOU THINK WOULD BE THE EFFECT ON THE BABY IF OTHERS SMOKED AROUND THE MOTHER WHILE SHE WAS PREGNANT? ___ 158
    - 0. No effect
    - 1. Physical defect
    - 2. Mental retardation or developmental delay
    - 3. Miscarriage or low birth weight
    - 4. Cancer
    - 5. Other (specify): ____________________ 159–174
    - 9. Do not know / No answer

27. DOES THE GOVERNMENT HAVE ANY POLICY TO BAN SMOKING IN PUBLIC PLACES? ___ 175
    - 0. No
    - 1. Yes
    - 9. Do not know / No answer

28. ARE THERE ANY EDUCATION PROGRAMS TO HELP PEOPLE STOP SMOKING? ___ 176

 0. No
 1. Yes
 9. Do not know / No answer

29. WHERE HAVE YOU SEEN OR HEARD INFORMATION ABOUT STOPPING SMOKING? ___ 177

 0. No policy or education program
 1. Billboards or posters in public places
 2. Television or radio announcements
 3. Newspaper or magazine announcements
 4. From doctors or other health care workers
 5. Other (specify): ____________________ 178–193
 9. Do not know / No answer

30. DOES THE GOVERNMENT HAVE ANY POLICY TO STOP PEOPLE FROM SMOKING AROUND PREGNANT WOMEN? ___ 194

 0. No
 1. Yes
 9. Do not know / No answer

31. ARE THERE ANY EDUCATION PROGRAMS TO TEACH PEOPLE TO STOP SMOKING AROUND PREGNANT WOMEN? ___ 195

 0. No
 1. Yes
 9. Do not know / No answer

32. WHERE HAVE YOU SEEN OR HEARD INFORMATION ABOUT STOPPING SMOKING AROUND PREGNANT WOMEN? ___ 196

 0. No policy or education program
 1. Billboards or posters in public places
 2. Television or radio announcements
 3. Newspaper or magazine announcements
 4. From doctors or other health care workers
 5. Other (specify): ____________________ 197–213
 9. Do not know / No answer

33. DID YOU TRY TO STOP YOUR HUSBAND FROM SMOKING AT HOME WHEN YOU WERE PREGNANT? ___ 214

 0. No
 1. Yes
 9. Husband does not smoke at home / No answer

34. DO YOU THINK PEOPLE SMOKING AROUND BABIES AND CHILDREN HAS ANY HEALTH EFFECT ON BABIES AND CHILDREN? ___ 215

 0. No
 1. Yes
 9. Do not know / No answer

35. WHAT DO YOU THINK WOULD BE THE EFFECT ON BABIES AND CHILDREN IF OTHERS SMOKED AROUND THEM?

0. No effect
1. Lung problems
2. Ear problems
3. Brain problems
4. Stunted growth
5. Cancer
6. Other problems (specify): ____________________
9. Do not know / no answer

___
216

217–232

What was your weight when you first became pregnant?

__________ kilograms

___ ___ . ___
233 234 235

THESE ARE ALL THE QUESTIONS I HAVE. THANK YOU FOR YOUR TIME.

[END OF INTERVIEW]
